# Mechanochemically Assisted Coal Fly Ash Conversion into Zeolite

**DOI:** 10.3390/ma15207174

**Published:** 2022-10-14

**Authors:** Ewelina Grabias-Blicharz, Rafał Panek, Małgorzata Franus, Wojciech Franus

**Affiliations:** Department of Construction Materials Engineering and Geoengineering, Faculty of Civil Engineering and Architecture, Lublin University of Technology, Nadbystrzycka 40, 20-618 Lublin, Poland

**Keywords:** mechanical activation, mechanochemistry, fly ash, fly ash utilisation, zeolite, fly ash-based zeolites

## Abstract

Mechanically treated fly ash (FA) was utilised to provide Al and Si atoms for zeolite synthesis. A combination of mechanical fly ash activation and classical hydrothermal synthesis led to favourable dissolution of activated fly ash and improved crystallization of zeolites. The milling activation step induced structural changes in FA to promote its reactivity in alkaline solution. The conversion of milled FA into zeolite materials was finally completed in the second step, during hydrothermal synthesis. The effect of such factors as crystallization temperature, milling time, and solution conditioning were systematically studied. The physicochemical properties characterising the obtained zeolite materials were determined via particle size distribution (PSD), nitrogen adsorption–desorption, X-ray fluorescence spectroscopy (XRF), scanning electron microscopy (SEM), and powder X-ray diffraction (XRD). As a result, the best samples achieved a high degree of crystallinity and an extensive specific surface area of 292 m^2^/g, 87.4 m^2^/g, 41.9 m^2^/g for Na-X, Na-P1, and Na-A, respectively. The obtained results provide new and useful data for utilising fly ash resources and synthesising other practical zeolites through an innovative, mechanochemically assisted, and template-free approach.

## 1. Introduction

Renewed interest in mechanosynthesis over recent years has attracted scientific attention as a promising alternative to traditional synthesis routes and aims to rationalise previously unknown mechanisms. Traditionally, grinding is a way to induce mechanochemical reactions in which transport of energy and mass occurs with low solvent quantities or under solvent-free conditions [1]. IUPAC (International Union of Pure and Applied Chemistry) defines a mechanochemical reaction as a ‘chemical reaction that is induced by the direct absorption of mechanical energy (shearing, stretching, and grinding are typical methods for the mechanochemical generation of reactive sites)’ [2]. The bond creation and rupture also includes grinding and milling processes, increasing the area of contact through reducing the size of the reactants. The term ‘milling’ is generally reserved for high speed/speed processes (such as ball-milling), while ‘grinding’ is used in the case of low-energy ones, involving mortar and pestle. However, their use in mechanosynthesis makes the repeatability of grinding conditions difficult. Nowadays, commercially available milling devices, such as planetary ball mill, mixer (shaker) extruder, cryomill, etc., ensure good reproducibility of the synthesis parameters (grinding time, speed of rotation, and/or substrate fragmentation) [1,3].

Up to now, mechanochemistry has been used in different scientific and industrial fields—such as metallurgy; mineral processing; and the synthesis of carbon materials, graphene, MOFs (metal–organic frameworks), alloys, and diverse organic–inorganic hybrid nanomaterials [1,3]—or to follow the properties of ground fly ashes as an additive in construction materials [4,5,6]. Ball milling has also been recently utilised during the mechanochemically assisted synthesis of numerous zeolites, mainly high-silicon zeolites [3,7,8,9]. Nada et al. [3] produced mordenite and ZSM-5 zeolite via mechanochemical activation of chemical reagents. The procedure they outlined was without the use of any templates, crystal seeds, or solvents. They obtained crystalline zeolite products with a specific surface area of about 300 m^2^/g. Pan et al. [7], on the other hand, also obtained zeolite ZSM-5 based on mechanical processing of the starting reagents. However, the authors used kaolinite as the starting material for zeolite synthesis, without the addition of organic templates. Moreover, Pashkova et al. [8] obtained the zeolite type of SSZ-13. They carried out hydrothermal synthesis to obtain zeolite SSZ-13, which was preceded by mechanical treatment of the chemical reactants. Moreover, Ren et al. [9] proved that mechanical activation of chemical reactants followed by heating at 180 C leads to zeolite type ZSM-5.

In spite of this progress, the mechanosynthesis of zeolites is still quite understudied. Due to the unique properties of zeolites (skeleton types, chemical composition, porosity, and stability), the mechanochemistry of zeolites appears to be worthy of further investigation. The milling activation process leads to structural changes in the starting reagents to promote their reactivity for direct transformation into zeolite materials. The crystal structure characterising solid reagents is destroyed by grinding force; at the same time, water may be added in small amounts or, alternatively, it may also be released from the hydrated derived reagents. The addition or release of water may aid in the reactions that occur between materials, ultimately leading to the occurrence of target materials and intermediates [1].

Coal fly ash (CFA) is produced during the combustion of coal in power plants and thermal power plants, as well as a principal waste product obtained when combusting fossil fuels. Among waste materials, CFA has gained significant interest due to its increasing production, which is related to the growing demand for energy consumption. The main CFA producers include China (500 Mt) and India (140 Mt), as well as the USA and Europe (114 Mt) [10,11,12]. According to the “Circular Economy” concept, intensive efforts are being undertaken globally to increase CFA recycling, forming new materials characterised by industrial and economic value [10,13]. A variety of methods for reusing this likely toxic waste were proposed (including cement and concrete production [5,13], roads, asphalt mix filler [6], geopolymers [14,15,16], bricks [17], lightweight aggregate [18], and environmental applications [19,20]. Among many different methods of fly ash disposal, converting coal fly ash into zeolite appears to be of greatest benefit while supporting the growing global demands towards sustainability [11].

Zeolites are microporous, crystalline aluminosilicate materials with a well-established 3D structure. They are composed of tetrahedral silicon- and aluminium-oxygen TO4 (T = Si, Al), which are linked with each together via corners sharing O atoms and forming channels, chambers, and cages present in the crystal framework of zeolites. Zeolites may be grouped into synthetic and natural ones. Natural zeolites are mainly of volcanic and hydrothermal origin. They can be found in both metamorphic rocks or sedimentary formations [3,11,21]. Synthetic zeolites are produced by various methods, such as classical hydrothermal synthesis, fusion method, molten salts method, and microwave-assisted method [11,22,23,24,25,26]. These employ various chemicals, temperatures, and process times; it is possible to produce different zeolite types. The most widely employed method of zeolite preparation, i.e., hydrothermal synthesis, involves using chemical reagents or is based on waste materials, e.g., clays or fly ash. According to the conditions of the synthesis reaction (reaction time, temperature, substrate ratio), several zeolite types of different purity and properties may be produced from the derived substrates. Based on fly ash, gmelinite, cancrinite, chabazite, analcime, Na-P1, Na-A, Na-Y, Na-X, and sodalite zeolites can be produced via different routes. These zeolites have been used in various chemical industry areas, e.g., shape-selective catalysis ion exchange, separation, and adsorption [11,22,27,28]. Type A zeolite is formed by tetrahedrons that are joined together by double 4-membered rings. The pore diameter is determined by the eight-membered oxygen ring with diameters from 0.23 to 0.42 nm. The X-type zeolite is formed by tetrahedra linked together by 6-membered rings, forming a hexagonal prism. This arrangement of structural units forms a second type of chambers called supercells about 1.3 nm in diameter, which are, in turn, connected by four 12-membered rings about 0.74 nm in diameter. The NaP1 zeolite is dominated by the tetrahedron system, which forms a 4-membered ring, the rings combine to form 8-membered rings with dimensions of 0.31 × 0.45 nm and 0.28 × 0.48 nm, respectively [29]. To the best of our knowledge, there are a limited number of papers available on zeolite mechanochemically assisted synthesis [3,7,8] and preparation of zeolites by mechanochemistry is underdeveloped so far. Mechanosynthesis by definition is considered to be a more sustainable and environmentally friendly method than traditional hydrothermal synthesis, so it seems to be necessary to study in detail the mechanosynthesis of zeolites based on waste materials (fly ash) conversion.

This paper investigates the use of an innovative mechanochemically assisted, template-free, and rapid synthesis of zeolite-type materials, based on fly ash (FA) utilisation. Zeolite mechanochemically assisted hydrothermal synthesis yielded LTA—Na-A, GIS—Na-P1, and FAU—Na-X zeolites. Moreover, the phase evolution of fly ash and zeolite with increasing temperature and conditioning of the reaction solution was followed. This strategy provides a detailed approach to FA utilization at laboratory scale with fast, efficient, and organic template-free route.

## 2. Materials and Methods

### 2.1. Mechanical Activation of Fly Ash

**Figure 1:**
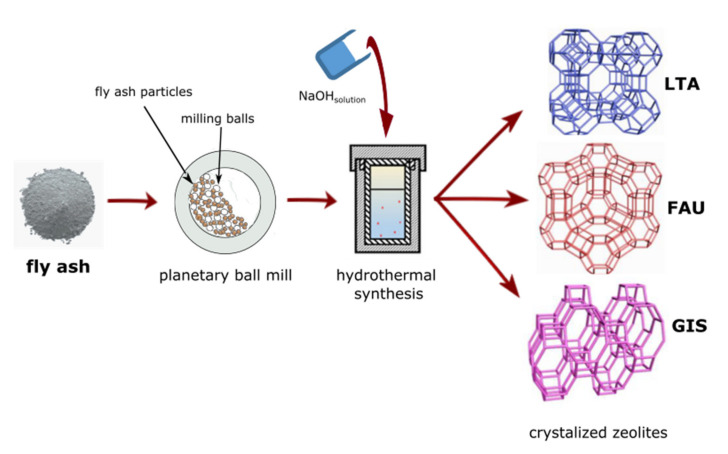
Graphical summary of zeolite phase formation by mechanochemically assisted coal fly ash conversion.

Fly ash (FA) powder was collected from Jaworzno Thermal Power Station (Jaworzno, Poland). As received FA (denote further as FA0) was milled using a Pulverisette-5 planetary ball mill (Fritsch, Germany), with the use of zirconia balls (20 mm) and 80 mL zirconia pot. The preparation of mechanically treated fly ash powders was carried out under the conditions below: milling time 1, 2, and 3 h at 400 rpm, without the use of any milling medium and with ball/powder ratio of 2:1 (Figure 1). Then, the obtained milled fly ashes were labelled as FA1, FA2, and FA3 (for 1, 2, and 3 h of milling time), respectively.

### 2.2. Conversion of Milled FA into Zeolite Type Materials

The Na-A, Na-P1, and Na-X zeolite materials (denoted hereinafter as Na-A, Na-P1, and Na-X, respectively) based on milled fly ash, were obtained via a series of experiments. Generally, mechanochemically treated fly ash in the amount of 10 g was subjected to mixing with the NaOH solution. NaOH of analytical grade was acquired from P. P.H. “Stanlab”, Lublin, Poland. The Na-A zeolite was synthesised using an additional source of Al, i.e., aluminium foil, to decrease the molar ratio of Si/Al. The considered zeolite types involved the process conditions shown below (for FA0, FA1, FA2, and FA3 fly ash samples), and based on the results of our previous experiments [30,31]:

- Synthesis of Na-A phase: 40, 60, 80, 100 °C; 3 M NaOH solution.
- Synthesis of Na-P1 phase: 80, 100, 120, 140 °C; 2 M NaOH solution.
- Synthesis of Na-X phase: 60, 80, 100, 120 °C; 3 M NaOH solution.

The hydrothermal conversion was conducted as a function of temperature (set individually for each zeolite phase) and solution conditioning. The resulting suspensions were subsequently transferred to an autoclave made of Teflon-lined stainless steel, and then placed for 24 h in the oven at different crystallization temperatures, according to the desired conditions. Following the hydrothermal synthesis, surplus alkaline solution was removed before rinsing the final material several times using distilled water followed by drying at 105 °C (Figure 1). The prepared zeolite materials were subjected to further analyses.

### 2.3. Materials Characterization

X-ray diffraction (XRD) was employed to determine the mineral composition, using X-ray diffractometer Panalytical X’pert PRO MPD (Multi-Purpose Diffractometer—Panalytical X’pert PRO MPD) (Panalytical, Eindhoven, The Netherlands) with a goniometer PW 3050/60 in the angle range of 5–65 2θ, with a step size of 0.02. The employed source of X-radiation corresponded to a copper lamp (CuKα = 0.154178 nm). The obtained diffraction data were processed by using the X’ Pert High Score software (version 4.1). Mineral phases were identified by means of PCPDFWIN PDF-2 release database ver. 1.30 formalized by JCPDS/ICDD.

A Quanta 250 FEG (Field Emission Gun) scanning electron microscope (SEM) by FEI (Hillsboro, OR, USA), including a chemical composition analysis system based on energy dispersion scattering EDS-EDAX, was used to investigate the morphology of the primary mineral components.

Energy dispersive X-ray fluorescence spectroscopy was employed to determine the chemical composition. It was performed using an Epsilon 3× ED-XRF spectrometer (Panalytical, Eindhoven, The Netherlands) with an X-ray tube, including a 50 kV Rh anode as the excitation source. Calculation of the obtained results was carried out with respect to the values of LOI (loss on ignition).

The low-temperature nitrogen adsorption/desorption isotherm was used to determine the textural parameters, characterizing the investigated materials. A Micromeritics ASAP 2020 (Micromeritics, Norcross, USA) analyser was used to perform measurements. Prior to the test, the outgassing of the samples was performed at a temperature of 250 °C for 24 h under high vacuum conditions. The standard Brunauer–Emmett–Teller (BET) equation was used to calculate the specific surface areas (SBET) for N adsorption data with relative pressure p/po range of 0.05–0.3.

The particle size distribution (PSD) characterizing milled and raw and FA samples was measured by means of the laser diffraction method on a Mastersizer 3000 analyser, with a HYDRO EV unit (Malvern, Panalytical, UK).

## 3. Results

### 3.1. FA Characterization

Table 1 shows the content of chemical elements in fly ash. The primary components of FA include SiO_2_ and Al_2_O_3_ with significant amounts of Fe_2_O_3_ (7.22%), CaO (3.05%), K_2_O (3.02%), and unburned carbon (3.29%), respectively. According to numerous studies [6,11,31,32], the tested fly ash belongs to class F due to oxide content, i.e., SiO_2_ + Al_2_O_3_ + Fe_2_O_3_ above 70%.

**Figure 2:**
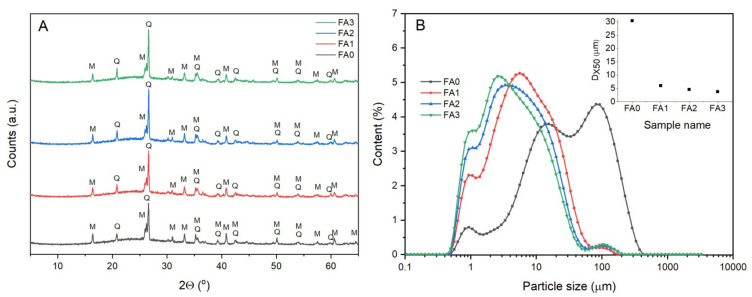
(**A**) XRD patterns, and (**B**) particle size distribution (PSD) (inset is the mean particle size Dx50) of raw and milled FA powders.

The mineralogy characterizing various phases of raw and milled FA samples is presented in Figure 2a. The identified primary crystalline phases include mullite and quartz. The XRD pattern indicates that amorphous aluminosilicate glass is present in substantial amounts (a broad characteristics hump between 18–30° 2θ). No visible changes in the peak positions, intensities, and shapes of the crystalline phases can be detected, as the mechanical treatment time is increased. Therefore, it may be stated that the structural changes, which could possibly impact the FA reactivity, are absent. However, mechanical treatment of FA significantly decreases the particle size for all milling conditions applied (Figure 2b and Table 2) (Dx10, Dx50 and Dx90 means that 10, 50 and 90% particles are smaller than measured value). There is a significant decrease in the values of Dx50 and Dx90 at all stages of milling. A slight reduction in diameter of particles can be seen until 3 h (FA3) of milling, when the particles begin interacting via weak Van der Waals-type adhesion forces [14,15]. Thus, ball milling may be ineffective in further reducing particle size. The FA milling results for 2 and 3 h (FA2 and FA3) differ minimally, with regard to particle size. One should bear in mind that with prolonged mechanical activation time, the milled particles will likely begin agglomerating easily due to substantial surface energy, which may cause an increase in particle size [33,34]. Hence, mechanical treatment that is too long is not economical in relation to energy efficiency and saving. Thus, it may be stated that the mechanical treatment of 2 h improved particle size reduction, contributed to fly ash homogenization, increased FA reactivity, and obtained high quality final (zeolite) materials [7]. The results obtained are in agreement with specific surface areas (S_BET_) of FA samples (Table 2). The raw FA has the lowest specific surface area, probably because cenospheres are present in the original FA. The S_BET_ of milled FA powder samples increased after mechanical treatment because of the occurrence of finer fraction. However, the S_BET_ value has no linear correlation with time of milling as a result of particle agglomeration.

**Figure 3:**
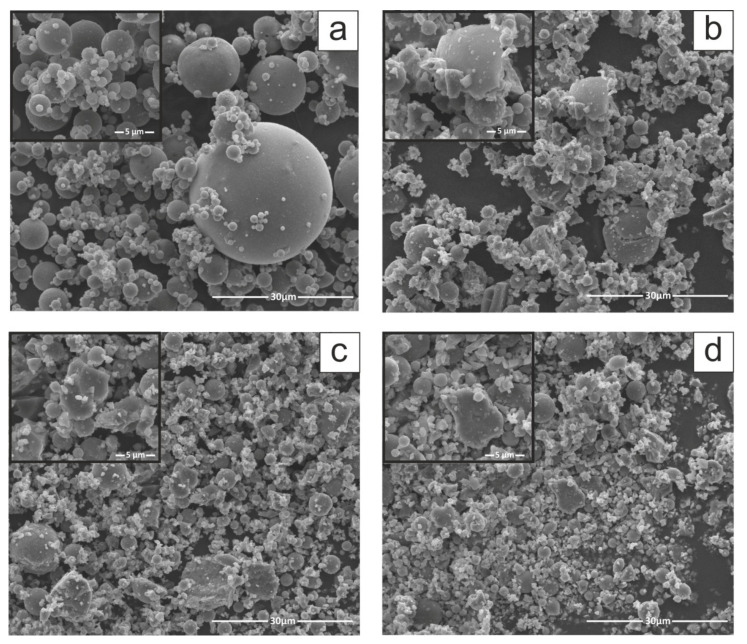
SEM micrographs of fly ash morphology (**a**) FA0 (raw), (**b**) FA1 (1 h of milling), (**c**) FA2 (2 h of milling) and (**d**) FA3 (3 h of milling) (magnification 4000, inset 16,000).

The comparison of raw and milled FA morphology was conducted by means of SEM micrographs to determine the changes in structure resulting from mechanical treatment. The raw fly ash SEM micrograph presented in Figure 3a shows that received FA particles are primarily spherical and granular, characterised by a relatively smooth surface texture and a wide range of particle size, as indicated by PSD (Figure 2b). The smooth surface exhibited by the as-received FA particles may occur because they are covered with amorphous glassy phase. This coating contributes to a smooth raw FA surface. The sphere-shaped particles observed in raw FA are generally formed when the inorganic fraction is suddenly cooled during coal combustion. Spherical FA particles are hollow or solid. The former, i.e., cenospheres, sometimes comprise smaller particles (plerosheres) [11,31,35]. The morphology of milled FA samples (Figure 3b–d) is greatly altered, whereas particle size decreases when milling time increases. The FA particles change shape from spherical to non-spherical as a result of comminution and agglomeration (Figure 2a–d). The particles that were originally spherical were crushed and attained irregular shape, as confirmed by numerous studies [14,33,34]. The coarser fraction of FA comprises spherical particles that break down into smaller sizes, and the particle shape irregularity is increased with milling. As a result of further milling, agglomeration of particles begins followed by deposition of loose fine particles on the surface of larger particles [15]. This transformation is in line with the PSD and SBET results for milled FA. Chu et al. [36] also confirmed high content of finer particles within the milled FA samples and indicated only a slight particle shape difference between various milling devices (attrition and vibration mill).

### 3.2. Effect of Ball-Milling and Crystallization Temperature

**Figure 4:**
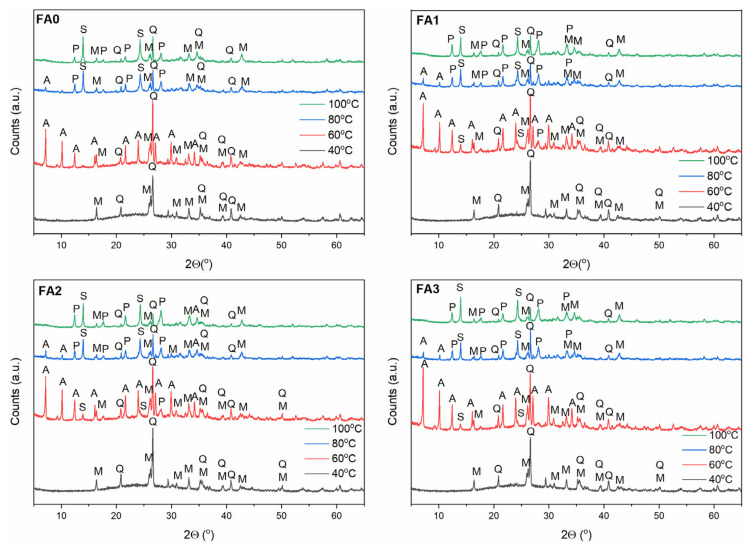
The effect of FA milling and temperature on FA conversion into Na-A zeolite phase. Clarifications: A—zeolite Na-A, M—mullite, Q—quartz, S—sodalite, P—zeolite Na-P1.

**Figure 5:**
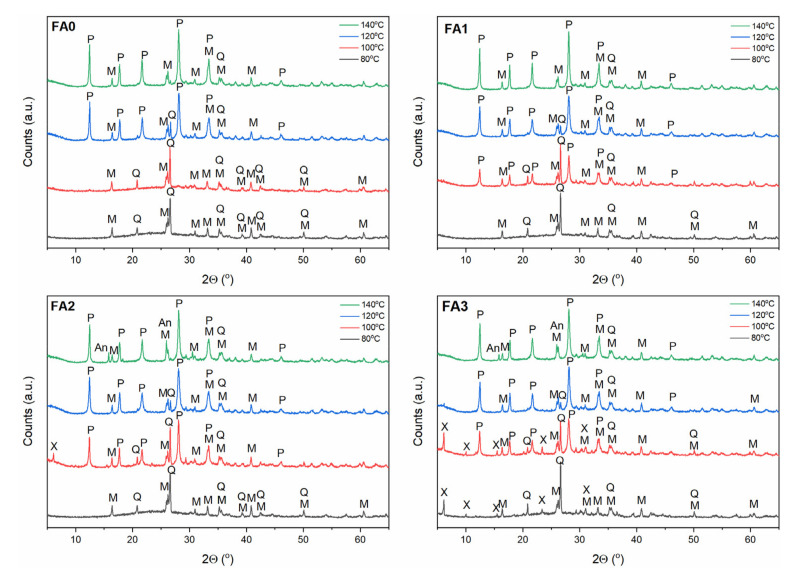
The effect of FA milling and temperature on FA conversion of into Na-P1 zeolite phase. Clarifications: X—zeolite Na-X, M—mullite, Q—quartz, An—analcime, P—zeolite Na-P1.

**Figure 6:**
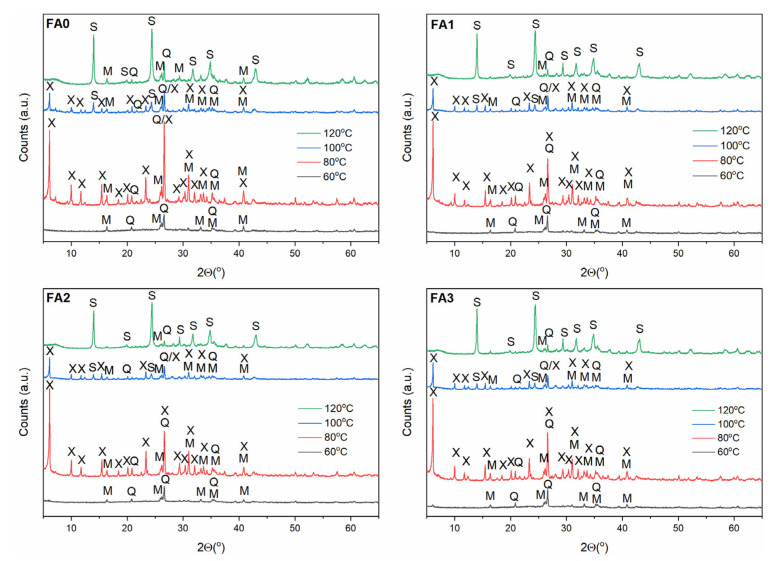
The effect of FA milling and temperature on FA conversion into Na-X zeolite phase. Clarifications: X—zeolite Na-X, M—mullite, Q—quartz, S—sodalite.

Zeolites are known to be highly sensitive to crystallization temperature due to their metastable behaviour [10]. They are thermodynamically unstable compared to dense structures, such as quartz and mullite present in FA. As such, according to the Ostwald rule of stages, zeolites transform from low- to high-density (i.e from less to more stable) structures [37]. To examine the FA milling time and crystallization temperature effect on FA conversion into zeolitic materials, experiments were carried out by altering the milling time and crystallization temperature, with a constant crystallization time (24 h). Hence, the crystallization temperature conditions were selected individually for each zeolite type based on preliminary studies and literature data [7,11,32,38] and milling times were 0, 1, 2, and 3 h, as presented in Section 2.1 and Section 2.2. The XRD patterns of the as-received zeolitic materials are depicted in Figure 4, Figure 5 and Figure 6. The XRD data (Figure 4, Figure 5 and Figure 6) reveal that the insolubility of fly ash powders in NaOH solution was greatly enhanced via mechanical treatment, owing to much smaller particles of the reactive glassy and crystalline phases and narrower particle size distribution in the ball-milled FA sample than untreated one (as indicated in Section 3.1) [14]. It is also known that Na^+^ ions stabilize the sub-micron building units of the forming crystal structure of zeolites. In addition, hydrated Na^+^ cations constitute a structure directing agent substituting the organic template, and a charge balancing agent [22]. The OH^−^ ions, as a very strong mineralizing agent, can induce and accelerate the dissolution of solid fly ash into gel, as well as the gel phase conversion to liquid phase. Furthermore, high-energy ball milling provided a sufficient amount of energy, causing a significant rupture of external Si-O/Al-O bonds in FA particles. After milling, the crushed FA particles were treated with NaOH solution, and then the reaction mixture was submitted to hydrothermal conditioning. Sodium hydroxide solution gradually dissolved the aluminosilicate glass in FA. Loosely bonded Si-O, as well as Al-O surface species of the fly ash particles, could easily react with NaOH solution to form zeolite phases, which was due to the fact that the larger surface area of finer particles were exposed to the alkaline solution by ball milling activation. Silicates and aluminates were transferred into gel via polycondensation reactions. Finally, the dissolved amorphous phase transformed into crystalline phases on the surface of FA particles, as well as the nucleation process of zeolite structures beginning [7]. The vast majority of milled fly ash components were converted into zeolites at the relevant temperature for each zeolite type (Figure 4, Figure 5 and Figure 6).

As indicated in the Section 2.2, different process conditions were applied to investigate the influence of temperature and mechanical treatment on the FA transformation into individual zeolite phases (the corresponding FA samples were designated as FA0, FA1, FA2, and FA3 for 0, 1, 2, and 3 h of milling). In the case of newly formed metastable Na-A-type zeolite (Figure 4), its structural transformations to the more stable and denser sodalite can be observed with increasing temperature (within the temperature range 40–100 °C). When the crystallization temperature reached 60 °C, the Na-A zeolite constituted the primary crystalline phase, accompanied by minor sodalite, quartz, and mullite phases. As the temperature increased above 60 °C, the main peak of sodalite (d_hkl_ = 3.65) continuously intensified, with a simultaneous decrease in the Na-A zeolite peaks (FA0–FA3, Figure 4). It is possible to explain this phenomenon by accounting for the metastability characterizing the Na-A zeolite phase. Na-A zeolites underwent gradual decomposition with increasing reaction temperature, contributing to liquid phase supersaturation with aluminosilicate anions, thus, enabling the formation of sodalite nuclei. Hence, crystallization at a temperature of 60 °C was favourable for the synthesis of Na-A zeolite with good crystallinity. On the contrary, Na-P1 zeolite phase (Figure 5) did not evolve to sodalite phase (in the temperature range 80–140 °C), but at 140 °C, analcime started to occur alongside Na-P1 phase, for FA2 and FA3. The finer fraction of FA2 and FA3, elevated synthesis temperature up to 140 °C and low NaOH concentration (2 M) may have been responsible for the formation of analcime as a more stable and denser phase, compared to sodalite and Na-P1. In the case of Na-X zeolite type, Na-X zeolite was the dominant crystalline phase at 80 °C (Figure 6). With increasing temperature, Na-X zeolite decomposed gradually, making the formation of sodalite nuclei possible. Overall, the crystalline end products exhibited high sensitivity to FA mechanical activation and crystallization temperature. For the formation of the monozeolite phase, crystallization temperatures of 60 °C, 80 °C, and 120 °C are favourable for synthesizing Na-A, Na-X, and Na-P1 zeolite type crystals. Consistent with literature reports [10,37], sodalite is a competing phase to Na-A and Na-X zeolite type, while Na-P1 zeolite tend to follow the sequence Na-P1 → analcime, with the increase in temperature. FA milling treatment and temperature increase resulted in enhanced levels of both the nucleation and crystallization rate. Furthermore, hydrothermal treatment of FA powders resulted in forming more thermodynamically stable phases, such as sodalite and analcime [7,10,37].

### 3.3. Effect of Ball-Milling and Solution Conditioning

**Figure 7:**
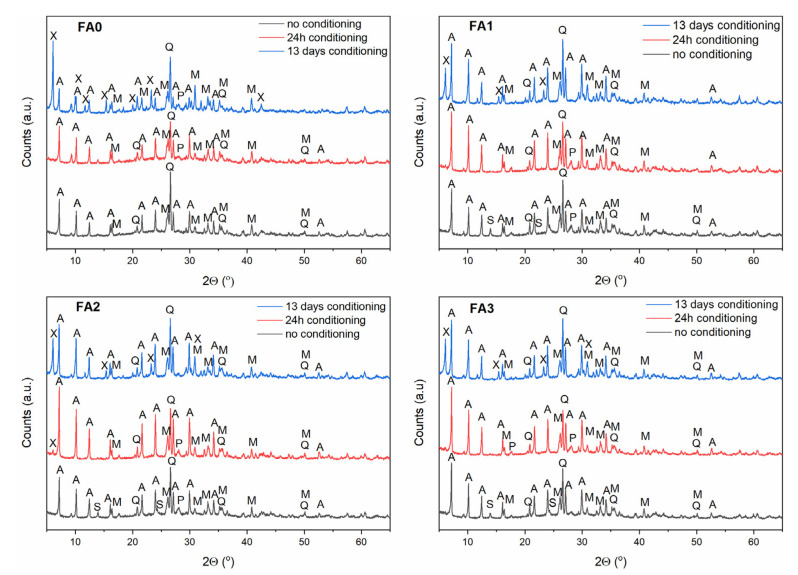
The effect of FA milling and solution conditioning on FA the conversion into Na-A zeolite phase. Clarifications: A—zeolite Na-A, M—mullite, Q—quartz, S—sodalite, P—zeolite Na-P1.

**Figure 8:**
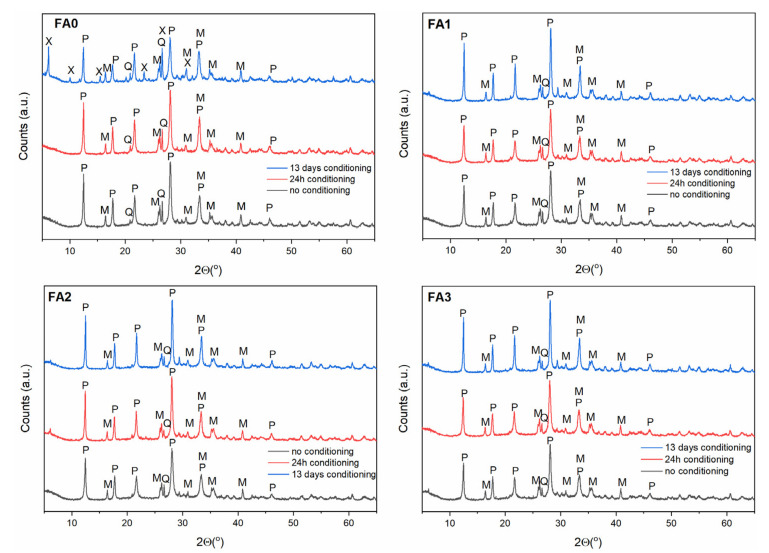
The effect of FA milling and solution conditioning on the FA conversion into Na-P1 zeolite phase. Clarifications: X—zeolite Na-X, M—mullite, Q—quartz, P—zeolite Na-P1.

**Figure 9:**
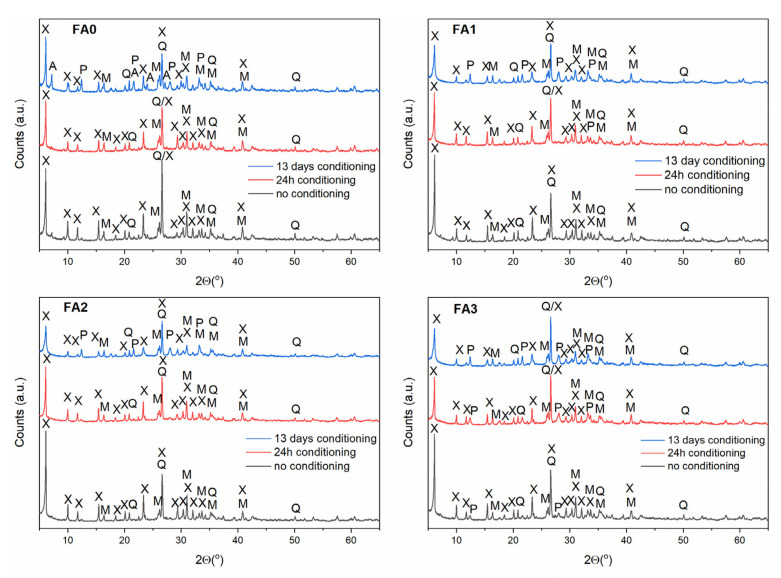
The effect of FA milling and solution conditioning on the FA conversion into Na-X zeolite phase. Clarifications: X—zeolite Na-X, M—mullite, Q—quartz, S—sodalite.

As evidenced in the previous section, the FA milling-activation step results in structural changes of the starting reagents to promote their reactivity in solution for the direct transformation into zeolitic materials. In order to make the FA dissolution in alkaline solution easier, a solution conditioning step is desirable, in addition to increasing the temperature [35,39,40,41,42]. Based on the results presented in Section 3.2, the FA conversion products were examined after 0 h, 24 h and 13 days of solution conditioning (Figure 7, Figure 8 and Figure 9) and under hydrothermal conversion at 60, 80, and 120 °C for Na-A, Na-P1, as well as Na-X-type zeolites, respectively. As far as the Na-A phase is concerned (Figure 7), the X-ray patterns revealed that Na-A zeolite was the primary crystalline phase, accompanied by minor fly ash impurities at no conditioning and 24 h conditioning time. After 13 days of solution conditioning, for FA0, Na-X was the dominant phase, alongside weakly formed Na-A, whereas for FA1, FA2, and FA3, Na-X and Na-A co-occurred as competing phases. When the influence of solution conditioning on FA conversion into the zeolite Na-P1 was examined (Figure 8), no marked differences regarding the quality of the crystalline end products were found for FA1, FA2, and FA3. Only in the case of FA0, after 13 days of solution conditioning, Na-X and Na-P1 appeared as competing phases. Moreover, a negative effect of solution conditioning time on FA conversion into zeolite Na-X was noted (Figure 9). Simultaneously, with prolonged solution conditioning, minor competing phases, Na-P1 and Na-A (for FA0), were identified. The solution-conditioning step seems to be desirable to increase the amount of Al^3+^ and Si^4+^ extracted from FA into the solution of alkaline substrate and required especially for Na-A zeolite crystal growth. In most cases studied, the products obtained were mixtures of zeolites and FA residues, because mullite and quartz remained partially undissolved under the conditions applied. On the whole, for the applied synthesis conditions, 24 h of solution conditioning proved to be most effective in terms of milled FA conversion into Na-A-type zeolite, whereas the non-conditioning of the solution seems to be positive for forming Na-P1 and Na-X type zeolites.

### 3.4. Characterization of FA-Derived Zeolitic Materials

Section 3.2 and 3.3 outlined that fly ash samples were successfully converted into zeolite phases with the assistance of mechanical activation. The wide hump (between 18 and 32 degrees 2θ) observed for the FA XRD patterns disappeared (Figure 2a), and the intensity of quartz and mullite peaks decreased markedly. As shown in Figure 4, Figure 5, Figure 6, Figure 7, Figure 8 and Figure 9, the intense sharp diffraction peaks show the occurrence of Na-A, Na-P1, and Na-X zeolites, as well as the dissolution of the partially crystalline phases and amorphous—both largely transformed during the zeolite formation process. The amorphous glass phase was observed to be the primary source of Al and Si atoms for zeolite crystallization because this phase is characterized by highest instability and solubility in FA. Mullite and quartz were noticed to be significantly less or non-reactive in alkaline solutions.

**Figure 10:**
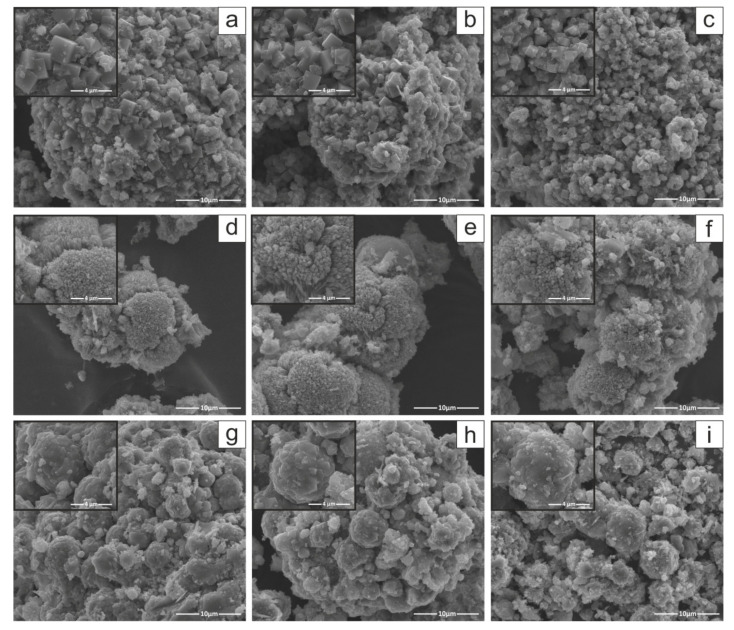
SEM images of zeolite products morphology, (**a**–**c**) Na-A, (**d**–**f**) Na-P1, (**g**–**i**) Na-X (magnification 8000, inset 30,000) for FA0, FA2, and FA3 as substrates, respectively.

Scanning electron microscopy (SEM) images were carried out only for the most prominent as-synthesized zeolite crystal products (Figure 10). Morphological observation of zeolite crystals allowed identifying individual representatives within this mineral group. SEM images (Figure 10) reveal a visible transformation of the crushed and spherical particles typical of raw and milled FA (Figure 3) into (i) cubic LTA-type zeolite crystallites for Na-A [32], (ii) lamellar aggregates with plate-like crystalline GIS structures for Na-P1 [35], (iii) quite irregular, sharp-edged FAU forms for Na-X [32]. According to the literature, the morphology presented is a typical morphology for Na-A, Na-P1, and Na-X type zeolites [11,31,32]. However, a tendency for smaller Na-A type zeolite crystals to grow with increasing milling time can be observed (Figure 10a–c). The SEM images also reveal that a residuum is present, corresponding to unreacted aluminosilicate glass.

**Figure 11:**
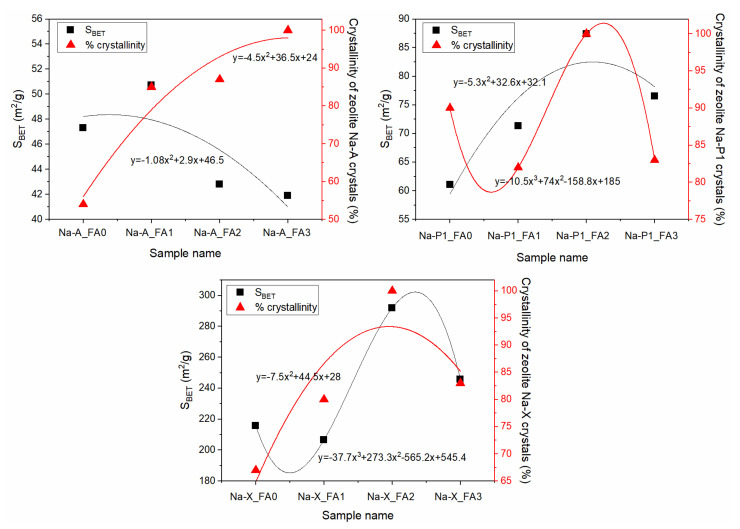
Relationship between specific surface area (S_BET_) and (%) crystallinity of the synthesized crystalline product of Na-A, Na-P1, and Na-X zeolites.

**Figure 12:**
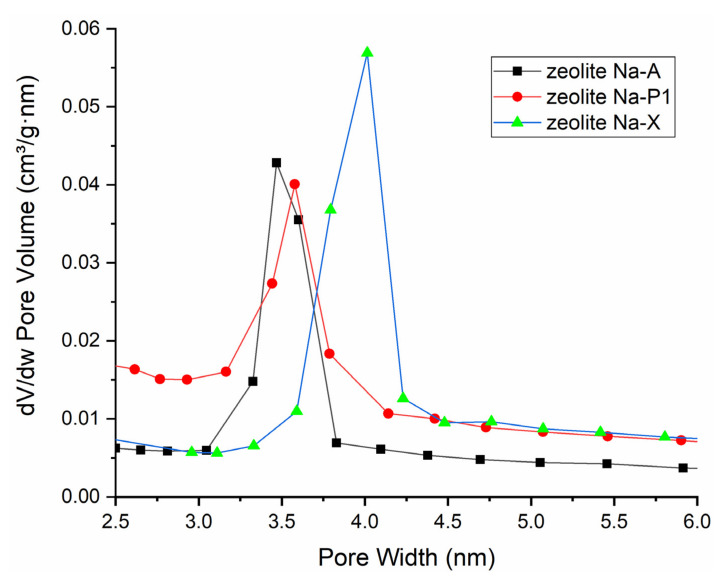
Pore size distribution for samples obtained under optimal conditions.

The equation below was used to calculate the quantitative content (% crystallinity) of every synthesised zeolite product:

(1) $$\%\;\mathrm{crystallinity}=\frac{A}{A_{0}}\times 100\%$$

where A is the average sum of peak areas of the seven main diffraction peaks while Ao is the greatest selected value of A that is the reference corresponding to 100% Equation (1) [22]. Relative crystallinity (%) was plotted as a function of specific surface area (S_BET_) and is presented in Figure 11. The crystallinity characterising the most crystalline zeolite products, Na-A_FA3, Na-P1_FA2, and Na-X_FA2, was determined as 100%. The residues of each synthesis reaction were mullite, quartz, and amorphous aluminosilicate glass from the synthesis mixture, which were presented at the XRD pattern (Figure 4, Figure 5, Figure 6, Figure 7, Figure 8 and Figure 9), as well as SEM images (Figure 10). In the case of the Na-P1 zeolite (Figure 11), the lack of FA milling yielded a zeolite material with a crystallinity of 90% and specific surface area of 61.1 m^2^/g. The first hour of milling resulted in the reversal of this relationship, i.e., increased specific surface area to 71.3 m^2^/g and a simultaneous decrease in crystallinity to 82%. Prolongation of fly ash milling time to 2 h increased both parameters (S_BET_ and % crystallinity) and obtained zeolite material containing almost 100% of Na-P1 phase with specific surface area of 87.4 m^2^/g. In contrast, further increasing the fly ash milling time to 3 h decreased both parameters, resulting in a material with similar parameters as the 1 h milling (3 h milling: 75 m^2^/g and 83%). For the zeolite material Na-X (Figure 11), for milling times of 0 h and 1 h, we observed an increase in crystallinity from 67 to 80% and a decrease in specific surface area from 217 to 206 m^2^/g, which is the inverse of that for Na-P1, at the same milling times. Increasing the milling time to 2 h, as in the case of the Na-P1 zeolite, resulted in a material with the highest crystallinity around 100% and the highest specific surface area of 292 m^2^/g. In contrast, extending the milling time to 3 h resulted in a reduction in both parameters to 83% for crystallinity and 246 m^2^/g for specific surface area. However, for Na-A zeolite, as the milling time increased, the crystallinity correlated inversely with S_BET_. Consistent with the XRD, SEM, and crystallinity results, the specific surface area (S_BET_) of the zeolite products (Figure 11) indicates that the optimal FA milling time is 2 h for Na-P1 and Na-X type zeolites. The case of Na-A-type zeolite is quite different. As the milling time of FA increases, the zeolite crystals obtained are smaller and smaller, with a tendency towards the formation of nanocrystals. S_BET_ values decrease with increasing FA milling time for Na-A zeolite. Furthermore, Na-A zeolite exhibits a three-dimensional cubic structure, as well as pore diameter of 3.5–4 Å [43] (Selim et al., 2017), so the channels of the Na-A zeolite can be partially blocked by a nitrogen molecule with the kinetic diameter of 3.64 Å. The lower S_BET_ values for both Na-A-FA2 (42.8 m^2^/g) and Na-A-FA3 (41.9 m^2^/g) zeolites are probably related to the smaller particle sizes and their higher tendency to agglomerate. However, on the basis of the combined XRD, SEM, and S_BET_ results, it may be concluded that for the 3 h milled fly ash, the highest conversion rate of FA into Na-A type zeolite was obtained. Pore size distribution for samples obtained under optimal conditions is presented in Figure 12. In general, the optimal conditions for mechanochemically assisted synthesis of the monomineral zeolite phases are as follows (Table 3): 3 h of FA milling, 3 M NaOH, 24 h of solution conditioning, and 60 °C for NaA; 2 h of FA milling, 2 M NaOH, no solution conditioning, and 120 °C for NaP1; 2 h of FA milling, 3 M NaOH, no solution conditioning, and 80 °C for NaX.

## 4. Conclusions

This study presents new insight into the influence of the mechanical activation of FA on the zeolite crystallization process, offering a prospective method of controlling the final crystal morphologies (as summarized in Figure 1). The mechanical treatment of fly ash results in increased specific surface area, finer particle size, and better availability of FA reactive components to hydrothermal conversion. During ball milling, the FA particles were broken into finer fractions by continuous friction, however, too long mechanical treatment is considered to be not economical in regard to energy efficiency and saving. SEM images and XRD patterns reveal that mechanical activation greatly influenced the zeolite crystallization process, as well as the crystalline end products (Na-A, Na-P1, and Na-X type of zeolites). The increase in FA milling time causes the decrease in crystal size of Na-A zeolite type with higher crystallinity, which may subsequently enhance the zeolite performance for specific applications. A mechanochemically assisted synthesis method can help rationally tailor the final crystal structure of zeolites and their properties without losing phase purity. Moreover, this innovative and comprehensive approach opens up new directions for synthesising zeolites, as well as other materials, including nanomaterials and functional materials. Moreover, extending the utilisation of fly ash resources or alternative difficult-to-manage wastes into other practical zeolite materials can be considered a process that is sustainable and may contribute to circular economy. To sum up, the optimum conditions for the mechano-assisted synthesis of fly-ash based zeolite phases are as follows: 3 h of FA milling, 3 M NaOH, 24 h of solution conditioning, and 60 °C for NaA; 2 h of FA milling, 2 M NaOH, no solution conditioning, and 120 °C for NaP1; 2 h of FA milling, 3 M NaOH, no solution conditioning, and 80 °C for NaX.

## Figures and Tables

**Table 1:** Chemical composition of FA Jaworzno (%).

Component	Content (%)
Na_2_O	0.49
MgO	0.95
Al_2_O_3_	25.80
SiO_2_	51.64
P_2_O_5_	1.76
SO_3_	0.66
K_2_O	3.02
CaO	3.05
TiO_2_	1.85
Fe_2_O_3_	7.22
Loss on ignition (LOI)	3.29

**Table 2:** Change in specific surface areas (S_BET_) and particle diameters of FA in relation to milling time.

FA Features	FA0	FA1	FA2	FA3
Dx90 (µm)	146.0	23.9	19.6	17.6
Dx50 (µm)	30.5	6.0	4.6	3.8
Dx10 (µm)	4.4	1.3	1.1	1.0
S_BET_ (m^2^/g)	3.88	5.56	6.75	5.24

**Table 3:** Crystallinity, specific surface area (S_BET_), and average pore sizes (D_av_) for samples obtained under optimal conditions.

Sample	Crystallinity [%]	S_BET_ [m^2^/g]	D_av_ [nm]
NaA	100	42	5.71
NaP1	100	87	7.16
NaX	100	292	9.16

## Data Availability

The data presented in this study are available on request from the corresponding author.
